# P-770. Impact of parallel use of low-complexity automated nucleic acid amplification tests and urine lateral flow lipoarabinomannan assays to detect tuberculosis in people living with HIV

**DOI:** 10.1093/ofid/ofae631.965

**Published:** 2025-01-29

**Authors:** Dana Hassneiah, Aliya Moreira, Bada Yang, Mathias Weis Damkjaer, Laura Olbrich, Stephanie Bjerrum, Maunank Shah, Ruvandhi Nathavitharana

**Affiliations:** Beth Israel Deaconess Medical Center, Boston, Massachusetts; Beth Israel Deaconess Medical Center, Boston, Massachusetts; University Medical Center Utrecht, utrecht, Utrecht, Netherlands; University of Southern Denmark, Odense, Syddanmark, Denmark; LMU University Hospital Munich, Munchen, Hamburg, Germany; University of Southern Denmark, Odense, Syddanmark, Denmark; Johns Hopkins, Baltimore, MD; Beth Israel Deaconess Medical Center, Boston, Massachusetts

## Abstract

**Background:**

Tuberculosis (TB) is the leading cause of death in people living with HIV (PWH). Missed and delayed TB diagnoses in PWH lead to worse outcomes. The World Health Organization (WHO) recommends the use of low-complexity automated nucleic acid amplification tests (LC-aNAAT) as the initial diagnostic test for TB and urine lateral flow lipoarabinomannan assays (LF-LAM) to assist with TB diagnosis in PWH. This systematic review assesses the impact of parallel use of respiratory sample LC-aNAAT and urine LF-LAM in PWH on patient-important outcomes.

**Table 1.**
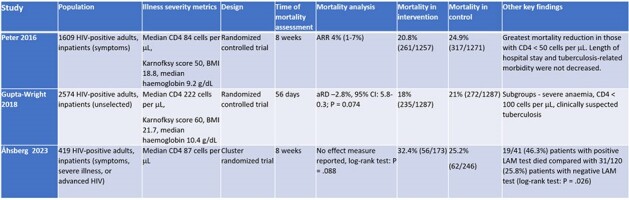
Comparison of mortality in randomized trials evaluating interventions that included respiratory LC-aNAATs and urine LF-LAM in adult inpatients with HIV .

**Methods:**

We searched multiple databases up to 3 November 2023. Eligible studies were randomized trials that allocated PWH to parallel LC-aNAAT and LF-LAM testing or LC-aNAAT alone or LF-LAM alone. We used a standardized form to extract data on mortality, proportion diagnosed with TB, proportion treated with TB and time to diagnosis and treatment. We assessed study quality using Cochrane Risk of Bias tools. Outcome measurement used random-effects meta-analysis to estimate pooled risk ratios with 95% confidence intervals.

**Figure 1.**
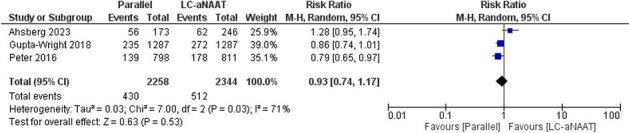
Impact of interventions that included respiratory LC-aNAATs and urine LF-LAM on mortality in adult inpatients with HIV

**Results:**

After 457 articles were screened, three randomized trials were included, all of which were conducted in inpatient settings in sub-Saharan Africa, with 4602 adult participants (Table 1). Risk-of-bias was low. The effect of parallel testing with respiratory LC-aNAAT and urine LF-LAM on mortality in PWH at eight weeks compared to testing with LC-aNAAT alone was uncertain (risk ratio 0.93; 95% CI 0.74 to 1.17) (Figure 2). Parallel testing may increase the proportion of patients with a confirmed TB diagnosis (pooled RR: 3.06, 95% CI 1.82 to 5.16) (Figure 3) and the proportion treated for TB (pooled RR: 1.47, 95% CI 1.25 to 1.73) (Figure 4) compared to LC-aNAAT alone. Time-to-treatment was shorter for the parallel testing group compared to the LC-aNAAT group in two trials and similar in the third.

**Figure 2.**
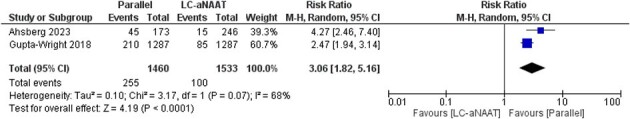
Impact of interventions that included respiratory LC-aNAATs and urine LF-LAM on the proportion of adult inpatients with HIV and with a confirmed tuberculosis diagnosis.

**Conclusion:**

In inpatient settings, the effect of parallel use of LC-aNAAT and LF-LAM on mortality in PWH at eight weeks was uncertain. Parallel testing may increase the proportion of PWH diagnosed with TB and treated for TB. There was no data on children or outpatient settings. Evidence is limited by heterogeneity in implementation approaches in included trials and contextual differences that included the effects of the COVID pandemic on one trial.

**Figure 3.**
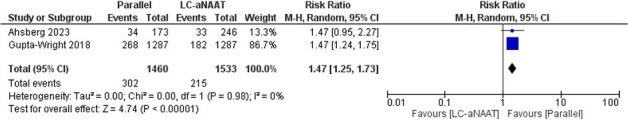
Impact of respiratory LC-aNAATs and urine LF-LAM on the proportion of adult inpatients with HIV treated for tuberculosis.

**Disclosures:**

**Laura Olbrich, PHD candidate**, Cepheid: received Xpert MTB/Rif ultra cartridges for free **Maunank Shah, MD, PhD**, Scene Health: Royalties for license

